# Thank you to our reviewers of 2023

**DOI:** 10.1002/epi4.12898

**Published:** 2024-01-23

The Editors of *Epilepsia Open* are very grateful to our Associate Editors and editorial board members as well as the following individuals for reviewing manuscripts in the past year. We are deeply appreciative of their insight, time, and effort.

Aristea S. Galanopoulou

Editor‐in‐Chief, *Epilepsia Open*

Dong Zhou

Deputy Editor, *Epilepsia Open*

***≥6 reviews, **≥3–5 reviews, *1–2 reviews

Eishi Asano***

Giulia Sofia Cereda***

Massimo Cossu***

Luca De Palma***

Gianluca D'Onofrio***

Giorgia Giussani***

Bruce Hermann***

Martin Holtkamp***

Stanislas Lagarde***

Simona Lattanzi***

Vineet Punia***

Mark Quigg***

Andreas Schulze‐Bonhage***

Vicente Villanueva***

Taylor Abel**

Action Amos**

Stéphane Auvin**

Sara Baldassari**

Carmen Barba**

Melissa Barker‐Haliski**

Robert Bollo**

Christian Brandt**

Andreas Brunklaus**

Jeffrey Buchhalter**

Petia Dimova**

Maurizio Elia**

Colin Ellis**

Giovanni Falcicchio**

Rimma Gamirova**

Elena Gardella**

Barry Gidal**

Jean Gotman**

Wang Hao**

Hans Hartmann**

Lawrence Hirsch**

Karl Klein**

Eric Kossoff**

Naoto Kuroda**

Lieven Lagae**

Wolfgang Löscher**

Zhengxiang Luo**

Emma Macdonald‐Laurs**

Theodor May**

Tanya McDonald**

Jesús‐Servando Medel‐Matus**

Victoria Morgan**

Solomon Moshe**

Heidi Munger Clary**

Kenneth Myers**

Andrew Neal**

Christos Papadelis**

Heath Pardoe**

Martina Perinelli**

M. Scott Perry**

Ahmed Raslan**

Antonella Riva**

Tristan Sands**

Andrea Santangelo**

Harvey Sarnat**

Evgenia Sitnikova**

Daichi Sone**

Ashwini Sri Hari**

Venus Tang**

Roland Thijs**

Pedro Viana**

Laurent Villard**

Randi von Wrede**

Bernd Vorderwülbecke**

Xuefeng Wang**

Zhong Wang**

Shennan Weiss**

Andrea Accogli*

Sophie Adler*

Umberto Aguglia*

Timothy Ainger*

Naoki Akamatsu*

Cigdem Akman*

Ozlem Akman*

Amza Ali*

Mohammad Daud Ali*

Alison Anderson*

Klara Andersson*

Ebru Apaydın‐Dogan*

Johan Arends*

Ali Asadi‐Pooya*

Etienne Audinat*

Gewalin Aungaroon*

Jerome Aupy*

Selma Aybek*

Ganna Balagura*

Alice Ballerini*

Fiona Baumer*

Sallie Baxendale*

Sándor Beniczky*

Mark Bennett*

Felix Benninger*

Gregory Bergey*

Christophe Bernard*

Federica Bertaso*

Astrid Bertsche*

Isabelle Beuchat*

Elisa Bianchi*

Valentina Biagioli*

Laura Bierhansl*

Leonilda Bilo*

Gretchen Birbeck*

Francesca Bisulli*

Marte Bjørk*

Leah Blank*

Davide Boido*

Ingo Borggrafe*

Ingrid Borthen*

Elie Bou Assi*

Farid Boumediene*

Dana Brabcová*

Kees Braun*

Francesco Brigo*

Walter Bruchhausen*

Katie Bullinger*

Jorge Burneo*

Alessia Cafaro*

Giuliana Cangemi*

Dezhi Cao*

Francesco Cardinale*

Melanie Carless*

Patrick Carney*

Jaime Carrizosa*

Fernando Cendes*

Mackenzie Cervenka*

Josephine Chan*

Lynn Chapieski*

Francine Chassoux*

Simela Chatzikonstantinou*

Chen Chen*

Ziyi Chen*

Michael Chez*

Nitish Chourasia*

Kon Chu*

Gaia Colasante*

Honor Coleman*

Erin Conrad*

Margherita Contento*

Antonietta Coppola*

Duccio Maria Cordelli*

Giorgio Costagliola*

Gregory Costain*

Beth Costine‐Bartell*

Joyce Cramer*

Claudia Cuccurullo*

Mark Cunningham*

Alice Dainelli*

Linda Dalic*

Undurti Das*

Kathryn Davis*

Carmen De Caro*

Marco de Curtis*

Antonella De Lillo*

Norman Delanty*

Jean Francois Desaphy*

Laxmikant Deshpande*

Alissa D'Gama*

Giancarlo Di Gennaro*

Ding Ding*

Raymond Dingledine*

Jakob Doerrfuss*

Michael Doherty*

Anna Doll*

Thinley Dorji*

Michael Doyle*

Blandine Dozieres‐Puyravel*

Anastasia Dressler*

Jacob Dubroff*

Mattias Duempelmann*

Sophie Dupont*

Anna Edelvik*

Monika Eisermann*

Tobias Engel*

Silke Ethofer*

Sara Eyal*

Firas Fahoum*

Antonio Falace*

Edward Faught*

Susanne Fauser*

Edoardo Ferlazzo*

Romano Ferruccio*

Guilherme Fialho*

Robert Fisher*

Niels Focke*

Emma Foster*

Thomas Freiman*

Jacqueline French*

Ayataka Fujimoto*

Hisako Fujiwara*

Lucia Fusco*

Kais Gadhoumi*

Athanasios Gaitatzis*

Antonio Gambardella*

Rita Garbelli*

Sara Gasparini*

Jay Gavvala*

Jozef Gecz*

Michael Gelfand*

Deepak Gill*

Lisa Gillinder*

Beatriz Giraldez*

Vadym Gnatkovsky*

Ethan Goldberg*

Sofia González‐Ortiz*

Howard Goodkin*

Jyotindra Goswami*

Bronwyn Grinton*

James Gugger*

Anne Hagemann*

Chellamani Harini*

Till Hartlieb*

Kenji Hashimoto*

Nicole Hawkins*

Hailan He*

Chloe Hill*

Gernot Hlauschek*

Gladys Ho*

Wenhan Hu*

Sheng‐Yao Huang*

Shaun Hussain*

Philip Iffland*

Katsumi Imai*

Kaleem Imdad*

Sara Inati*

Hamid Irannejad*

Naoum Issa*

Masaki Iwasaki*

Victoria Jackson*

Puneet Jain*

Damir Janigro*

Mubeen Janmohamed*

Floor Jansen*

Katrien Jansen*

Katrine Johannesen*

Erica Johnson*

Meredith Johnson*

Hennric Jokeit*

Nigel Jones*

Suchitra Joshi*

Csaba Juhasz*

Hoon‐Chul Kang*

Andres Kanner*

Jaideep Kapur*

Jennifer Kearney*

Sattar Khoshkhoo*

Michael Kinney*

Laura. Kirkpatrick*

Johanna Kissler*

Frank Kiwanuka*

Pavel Klein*

Alexandra Klotz*

Sookyong Koh*

Christian Korff*

Sanjeev Kothare*

Günter Krämer*

Gregory Krauss*

Hana Kubova*

Mathieu Kuchenbuch*

Mansur Kutlubaev*

Angela La Neve*

Fred Lado*

Suncica Lah*

Siew Tim Lai*

Joshua Larocque*

Dominique Leitner*

Johannes Lemke*

Camelia Lentoiu*

Antonio Leo*

Ilo Leppik*

Tally Lerman‐Sagie*

Gaetan Lesca*

Costin Leu*

Lin Li*

Yi Li*

Jianxiang Liao*

José Luiz Liberato*

Laura Librizzi*

Chemin Lin*

Sufang Lin*

Chang Liu*

Maria‐Leonor Lopez Meraz*

Elles Louw van der*

Joaquin Lugo*

Brian Lundstrom*

Luigi Maccotta*

Helio Machado*

Cosimo Maggiore*

Thendo Makhado*

Konstantin Makridis*

Federica Malerba*

Stephen Malone*

Michael Malter*

Raffaele Manni*

Carla Marini*

Bertrand Mathon*

Sara Matricardi*

Joanna Mattis*

Patrick May*

David McArthur*

James McNamara*

Kimford Meador*

Oriano Mecarelli*

Cameron Metcalf*

Andrew Michalak*

Mathieu Milh*

James Mills*

Berge Minassian*

Tonmoy Monsoor*

Barbara Mostacci*

Wenzhu Mowrey*

Marco Mula*

Naluca Mwendaweli*

John Mytinger*

Nael Nadif Kasri*

Lina Nashef*

Robert Nass*

Janak Nathan*

Chetan Nayak*

Clemens Neudorfer*

Marcus Ng*

John‐Paul Nicolo*

Giulia Nobile*

Lino Nobili*

Francesco Noe*

Douglas Nordli III*

Mahnaz Noroozi*

Jan Novy*

Ewan Nurse*

Terence O'Brien*

Karen Oliver*

Filiz Onat*

Tomonori Ono*

Francesca Operto*

Sandra Orozco‐Suarez*

Laila Ouchen*

Shahrbanoo Pahlevanynejad*

Eleonora Palma*

Andre Palmini*

Justyna Paprocka*

Jun Park*

Angelo Pascarella*

Dipan Patel*

Giada Pauletto*

Giovanni Pellegrino*

Page Pennell*

Chiara Pepi*

Roro Rukmi Windi Perdani*

Emilio Perucca*

Marta Piccioli*

Luciana Pimentel‐Silva*

Francesco Pisani*

Asla Pitkänen*

Tilman Polster*

Ofer Prager*

Alberto Preda*

Jan Pukropski*

Kurupath Radhakrishnan*

Nandhagopal Ramachandiran*

Georgia Ramantani*

Chaturbhuj Rathore*

Aylin Reid*

Jan Rémi*

Cristina Reschke*

Elisabeth Reus*

Joshua Richman*

Michele Rizzi*

Marian Roan*

Antonino Romeo*

Alessandra Rossi*

Jessica Rossi*

Karl Rössler*

Rob Rouhl*

Rejane Rua*

Theodor Rüber*

Stephan Rüegg*

Fergus Rugg‐Gunn*

Emilio Russo*

Iván Sánchez Fernández*

Josemir Sander*

Raman Sankar*

Rani Sarkis*

Shifteh Sattar*

Marcello Scala*

Frederic Schaper*

Helen Scharfman*

Katharina Schiller*

Natasha Schoeler*

Stephan Schuele*

Elisa Schuetz*

Elisa Schütz*

Leepile Sehularo*

Arjune Sen*

Tawfeeq Shekh‐Ahmad*

Kaili Shi*

Hiroshi Shigeto*

Jerry Shih*

Yael Shiloh‐Malawsky*

Hideaki Shiraishi*

Simon Shorvon*

Pierre Sicard*

Fong Si‐Lei*

Graeme Sills*

Hugh Simpson*

Gagandeep Singh*

Jaysingh Singh*

Suzette Smiley‐Jewell*

Elson So*

Jo Sourbron*

Richard Staba*

Mirja Steinbrenner*

Trevor Steve*

Jenny Stritzelberger*

Sven Stuiver*

Kenji Sugai*

Joseph Sullivan*

Rainer Surges*

Joji Suzuki*

Charles Szabo*

Pierre Szepetowski*

Maurizio Taglialatela*

Tracie Tan*

Xinjie Tan*

James Tao*

Olga Taraschenko*

Alexis Tarrada*

Laura Tassi*

Jeffrey Tenney*

Maria Thom*

Maoqiang Tian*

Xiaojuan Tian*

Steven Tobochnik*

Mario Tombini*

Eugen Trinka*

Marina Trivisano*

Melissa Tsuboyama*

Henna Tyynismaa*

Sara Uccella*

Tomas Uher*

Aycan Ünalp*

Lata Vadlamudi*

Kette Valente*

Gilles van Luijtelaar*

Anna Elisabetta Vaudano*

Sarah Velissaris*

Liana Veneziano*

Federico Vigevano*

Ana Vilan*

Maurizio Viri*

Tim von Oertzen*

Felix von Podewils*

Sarah von Spiczak*

Janelle Wagner*

Genna Waldman*

Katrin Walther*

Shuang Wang*

Yao Wang*

Amanda Saphira Wardani*

Samantha Weber*

Timothy Welty*

Benjamin Whatley*

Victoria Whiteley*

Robyn Whitney*

Toby Winton‐Brown *

Elaine Wirrell*

Juri‐Alexander Witt*

John Wolf*

Stefan Wolking*

Chong Wong*

Sheng Wong*

Ye Wu*

Kevin Xie*

Kedi Xu*

Elza Márcia Yacubian*

Yoshiaki Yamamoto*

Ruta Yardi*

Mirac Yildirim*

Lifei Yu*

Gaetano Zaccara*

Pia Zacher*

Ifrah Zawar*

Rina Zelmann*

John Zempel*

Chunqing Zhang*

Yaodong Zhang*

Honghua Zheng*

Maeike Zijlmans*

Sameer Zuberi*

